# Effects of Lycopene on Sheep Oocyte Maturation and Subsequent Parthenogenetic Embryo Development

**DOI:** 10.3390/antiox15060675

**Published:** 2026-05-27

**Authors:** Zhenghang Li, Wenjuan Zhao, Zihao Ma, Jiali Zhu, Shangya Deng, Yue Zhang, Weibin Zeng, Pengcheng Wan, Guangdong Hu

**Affiliations:** 1 College of Animal Science and Technology, Shihezi University, Shihezi 832003, China; 13894117372@163.com (Z.L.); 19131385019@163.com (Z.M.); 17339821283@163.com (J.Z.); dengshangya1234@163.com (S.D.); zykysha@163.com (Y.Z.); zwbdky@126.com (W.Z.); 2 Institute of Animal Husbandry and Veterinary Science, Xinjiang Academy of Agricultural Reclamation Sciences, Shihezi 832000, China; zwj-130@163.com; 3 National Key Laboratory of Genetic Improvement and Healthy Sheep Breeding, Shihezi 832000, China

**Keywords:** antioxidant, lycopene, oocyte, embryo, oxidative stress

## Abstract

Natural pigment lycopene (LYC), a carotenoid, possesses antioxidant, anti-apoptotic, anticancer, and immunoenhancing properties. During in vitro culture, this substance protects oocytes and early embryos from damage caused by reactive oxygen species (ROS), thereby enhancing the in vitro maturation (IVM) rate of oocytes and the developmental competence of early embryos. This study aimed to investigate the effects of supplementing different concentrations of LYC (0, 5, 10, and 15 μM) during in vitro culture of sheep oocytes and early embryos on their developmental competence. In contrast to the control group, the 5 μM LYC treatment group displayed a marked increase in the first polar body extrusion rate and the extent of cumulus cell expansion, as well as a significantly higher proportion of normal spindle assembly in sheep oocytes, but 15 μM LYC appeared to negatively affect oocyte maturation. Relative to all other experimental groups, the 5 μM LYC treatment group displayed significantly elevated rates of cleavage and blastocyst rate during early in vitro embryonic development. The levels of ROS in mature oocytes and early embryos were significantly decreased, whereas the GSH level was significantly elevated. Furthermore, LYC treatment significantly enhanced mitochondrial activity and markedly elevated the mitochondrial membrane potential (MMP) in mature oocytes and early embryos. Moreover, the total cell number of blastocysts was significantly increased. Moreover, in early embryos, the transcript levels of genes associated with both oxidative stress and apoptosis were favorably regulated. In conclusion, LYC supplementation boosted the rates of oocyte maturation and blastocyst formation in sheep, while elevating the developmental capacity of early embryos.

## 1. Introduction

The in vitro production (IVP) technology of sheep embryos, which combines oocyte pick-up (OPU) technology with in vitro maturation (IVM), in vitro fertilization (IVF), and in vitro culture (IVC) systems, not only helps in the conservation of endangered breeds but also opens up new avenues for sheep breeding through gene editing [1]. However, the efficiency of IVP remains constrained by the procedures of IVM, IVF, and IVC; therefore, continual refinement of the culture systems for these three stages is crucial for improving the quality of early embryonic development [2]. In recent years, the application of IVP technology in small ruminants has been steadily increasing. Consequently, optimizing the key culture systems within this technology is of paramount importance.

During IVM in the laboratory, immature oocytes are cultured in an environment designed to simulate in vivo conditions. Under these circumstances, oocytes are susceptible to damage from reactive oxygen species (ROS) during collection and culture, leading to oxidative stress that can ultimately impair their subsequent developmental competence [3]. Therefore, the balance between ROS and antioxidants is crucial for oocyte developmental competence [4]. Under physiological conditions, a balance is maintained between the pro-oxidant and antioxidant systems in mammalian cells [5]. However, excessive production of ROS can disrupt intracellular redox homeostasis and induce oxidative stress (OS) [6]. Previous studies have indicated that, under in vitro culture conditions, oocytes are susceptible to ROS-mediated signaling, which can destabilize the maturation-promoting factor (MPF) and lead to embryonic developmental arrest and reproductive disorders [7]. Furthermore, when intracellular ROS levels exceed the physiological range, they can induce germ cell depletion and accelerate ovarian aging [8]. Furthermore, ROS are generated by various subcellular organelles, with mitochondria serving as the primary source in mammalian cells [9].

To counteract the damage induced by excessive ROS, several exogenous antioxidants have been demonstrated to positively influence oocyte maturation and embryonic development, such as curcumin [10], leonurine [11], astragaloside IV [12], and melatonin [13]. LYC, a naturally occurring antioxidant predominantly found in tomatoes, exhibits potent antioxidant activity compared to other carotenoid antioxidants [14]. Based on its role in protecting against ROS-induced stress damage and mitigating inflammation, the natural compound LYC has been utilized in the prevention of cardiovascular diseases, the regulation of blood pressure, and the improvement of endothelial dysfunction [15]. Previous studies have demonstrated that LYC can alleviate damage in neuroblastoma cells exposed to H_2_O_2_ and reduce intracellular ROS levels [16]. Furthermore, supplementation of a specific concentration of LYC into ram semen extenders protects sperm against oxidative damage caused by ROS during the freeze-thaw process and significantly enhances post-thaw sperm motility and overall quality [17]. Moreover, the addition of a specific concentration of LYC to the in vitro culture medium led to a reduction in OS and extent of apoptosis in porcine embryos, thereby improving their developmental quality [18]. To date, the role of LYC on sheep oocyte maturation and early embryo development remains unexplored.

This study assessed the role of graded concentrations of LYC on sheep oocytes and early embryos by assessing intracellular glutathione (GSH), ROS levels, mitochondrial membrane potential, antioxidant capacity, and apoptosis-related gene expression. The results preliminarily demonstrate that LYC enhances oocyte maturation and early embryonic development. This provides a theoretical basis for optimizing the IVP system in the livestock industry.

## 2. Materials and Methods

### 2.1. Materials

Ovine ovaries were obtained from a licensed slaughterhouse for cattle and sheep located in Shayibake Village, Shihezi City. Fetal bovine serum (FBS) was purchased from Gibco (Waltham, MA, USA). MEM-NEAA (100×), GlutaMAX (100×), BME-EAA (50×), and sodium pyruvate (100 mM) were purchased from Thermo Fisher (Waltham, MA, USA). Bovine serum albumin (BSA) was purchased from GPC (Beijing, China). TCM199 basal medium was purchased from Procella (Wuhan, China). The Enhanced Mitochondrial Membrane Potential Assay Kit (JC-1), Reactive Oxygen Species Assay Kit, Tubulin Tracker Green, and Mito Tracker Red CMXRos were purchased from Beyotime (Shanghai, China). Follicle-stimulating hormone (FSH) for injection and luteinizing hormone (LH) for injection were purchased from Solarbio (Beijing, China). The RNAprep Pure Micro Sample Total RNA Extraction Kit was purchased from Tiangen (Beijing, China). The HiFiScript cDNA Synthesis Kit and 2× UltraSYBR Mixture were purchased from CWBIO (Beijing, China). LYC and monochlorobimane (chlorobimane) were purchased from MCE (Monmouth Junction, NJ, USA).

### 2.2. LYC Concentration Screening

A stock solution of LYC was prepared at a concentration of 2 mM using tetrahydrofuran (Aladdin, Shanghai, China) as the solvent. Working solutions with final concentrations of 5, 10, and 15 μM were subsequently prepared by dilution for experimental use. The concentration selection for this study was made with reference to the existing literature on various species [18,19,20]. In each experiment, the final THF concentration in all groups was below 0.5%, a concentration that does not affect cell growth [21]. The Negative Control (NC) group consisted of basic culture medium without the addition of LYC, but with the same volume of THF added as in the experimental group.

### 2.3. Collection and Maturation of Cumulus-Oocyte Complexes (COCs)

Ovaries were collected from healthy ewes aged 2–3 years and transported to the laboratory within 1–3 h in a thermos containing physiological saline maintained at 37.5 °C. Upon arrival, they were rinsed repeatedly with warm physiological saline and sterile physiological saline to remove surface debris and were subsequently transferred into mature oocyte collection medium at 37 °C. Ovarian follicles were incised with a surgical blade in egg collection fluid to release cumulus-oocyte complexes (COCs). COCs were collected under a stereomicroscope using a mouth-controlled pipette. Oocytes with at least two intact layers of cumulus cells were selected, which were then collected in oocyte collection media at 37 °C. The main components of the oocyte collection medium were TCM199 medium (500 mL), sodium heparin (0.025 g), gentamicin (0.025 g), and FBS (2%; vol/vol). Only oocytes displaying a uniform cytoplasm and enclosed by a minimum of three dense layers of cumulus cells were selected under a Nikon (SMZ800N, Tokyo, Japan) stereomicroscope for IVM. These selected COCs were thoroughly rinsed 3–4 times with pre-warmed IVM medium and allocated into four-well plates. IVM medium containing 0, 5, 10, and 15 μM LYC was supplemented with approximately 40 COCs, then the plates were cultured for 24 h in an incubator maintained at 38.5 °C, 5% CO_2_, followed by calculation of the maturation rate. All experiments were conducted in triplicate. The medium was TCM199 supplemented with GlutaMAX (100×, 200 mM), sodium pyruvate (100 mM), FSH (4 mg/mL), β-estradiol (1 mg/mL), gentamicin (1.6 mg/mL), LH (4 mg/mL), and 10% (vol/vol) FBS. Oocytes were cultured at 38.5 °C in a 5% CO_2_ incubator for 24 h. The expansion area of COCs in each group was measured using ImageJ software (version 1.54).

### 2.4. Parthenogenetic Activation (PA) and Embryo Culture

Following 24 h of IVM, the ovine oocytes were transferred to IVM medium containing 0.1% hyaluronidase and gently pipetted to completely remove the cumulus cells. Denuded oocytes were treated with 8% ethanol in IVC medium for 5 min. Following this, they were washed 2–3 times with fresh IVC medium and transferred to the parthenogenetic activation medium supplemented with 2 mM 6-DMAP (Sigma, St. Louis, MO, USA). After 4 h, the oocytes were washed three times with IVC medium to remove any residual 6-DMAP. Subsequently, early embryos were cultured for 7 days in four-well plates containing IVC medium supplemented with varying concentrations of LYC (0, 5, 10, and 15 μM) at 38.5 °C under 5% CO_2_. The cleavage rate was recorded at 48 h of culture, and the blastocyst rate was determined on day 7. The basal IVC medium consists of SOFJC supplemented with BSA, MEM-NEAA (100×), BME-EAA (50×), sodium pyruvate (100 mM), GlutaMAX (100×, 200 mM), gentamicin (1.6 mg/mL), CaCl_2_ (277 mM), and myo-inositol (277 mM).

### 2.5. Immunofluorescence Staining of α-Tubulin in Oocytes

Mature oocytes were fixed in immunostaining fixative for 30 min at ambient temperature. Following fixation, the oocytes were washed thoroughly three times with immunostaining wash buffer for 5 min each. The staining working solution was prepared by diluting Tubulin-Tracker Green to the appropriate concentration with immunostaining secondary antibody dilution buffer. The oocytes were then incubated in the staining working solution in the dark at ambient temperature for 30–60 min, after which three washes with immunostaining wash buffer for 5 min each. Finally, the mounted samples were visualized with a Nikon (AXR, Tokyo, Japan) laser scanning confocal microscope.

### 2.6. Detection of ROS and GSH Levels

The intracellular levels of ROS and GSH in mature oocytes and early embryos were assessed using the fluorescent probes DCFH-DA and monochlorobimane, respectively. Mature oocytes and early embryos from each group were then incubated in staining solution containing either 10 μM DCFH-DA for ROS or 10 μM Monochlorobimane for GSH. The incubations were carried out for 25 min in the dark at 38.5 °C under 5% CO_2_. After staining, the embryos were collected and washed three times in TCM199 1× medium, and then imaged using a Lecia (DMi8, Wetzlar, Germany) inverted fluorescence microscope. The green fluorescent images were analyzed with ImageJ software.

### 2.7. Detection of Mitochondrial Membrane Potential (MMP)

MMP in mature oocytes and early embryos was measured using the specific probe JC-1. To prepare the staining solution, 5 μL of JC-1 (200×) was mixed with 1 mL of JC-1 staining buffer. Randomly chosen early embryos were then incubated in this solution. They were cultured in an incubator at 38.5 °C in the dark. After 25  min, the embryos were collected and washed three times in TCM199 1× medium. Images were captured using a Lecia (DMi8) inverted fluorescence microscope. Red and green fluorescence images were analyzed using ImageJ software.

### 2.8. Detection of Mitochondrial Activity

Mitochondrial activity was measured using CMXRos. The CMXRos stock solution was diluted in IVM and IVC medium to prepare a 200 nM staining working solution. Randomly selected mature oocytes and early embryos were incubated in this solution for 25 min at 38.5 °C in the dark, followed by three washes with appropriate medium. Imaging was performed using a Lecia (DMi8) inverted fluorescence microscope. The fluorescence images were analyzed with ImageJ software.

### 2.9. Immunofluorescence (IF) Staining of Blastocyst

Day 7 blastocysts from each group were fixed with paraformaldehyde (PFA) for 30 min at ambient temperature. After fixation, the blastocysts were washed three times for 5 min each with Wash Buffer. The blastocysts were permeabilized with immunostaining permeabilization buffer for 30 min at room temperature. Subsequently, we washed them three times for 5 min each in Wash Buffer. They were subsequently washed three times for 5 min each in wash buffer and then blocked with blocking buffer for 4 h at ambient temperature. The early embryos from each group were then washed and stained with DAPI for 5 min. Finally, the mounted samples were visualized with a Nikon (AXR) laser scanning confocal microscope.

### 2.10. Real-Time Fluorescence Quantitative PCR

For each experimental group, 30–40 eight-cell embryos were randomly collected and polled. Total RNA was extracted using a micro-sample RNA kit, and cDNA was synthesized with a reverse transcription kit. The fluorescence quantitative reaction system was 20 μL, including 2 μL of cDNA, 10 μL of SYBR Green master mix, 0.8 μL of each forward and reverse primer, and 6.4 μL of RNase-free water. The PCR protocol was as follows: 95 °C for 5 min, then 45 cycles of 95 °C for 10 s, 58 °C for 30 s, and 72 °C for 32 s. All primers used in this study were designed with Primer 5.0 (Table 1). The relative mRNA expression was calculated using the 2^−ΔΔCt^ method, with β-actin as the internal reference gene.

### 2.11. Experimental Design

In experiment 1, a total of 399 oocytes across three independent replicates were cultured for 24 h in IVM medium containing 0, 5, 10, or 15 μM LYC to compare the effects of various LYC concentrations on oocyte IVM. Subsequently, the developmental competence of each LYC-treated group was assessed by the PB1 extrusion rate and growth and expansion of COCs. In experiment 2, a total of 150 oocytes was used in three independent replicates. To assess the antioxidative effect of LYC on IVM oocytes, intracellular ROS levels, GSH levels, and JC-1 levels in oocytes treated with the control and the optimal concentration of LYC. In experiment 3, a total of 588 blastocysts was used in three independent replicates. To investigate the effect of various concentrations of LYC on the rate of blastocyst and cleavage rate in PA embryos. In experiment 4, a total of 102 Day-5 PA embryos from three independent replicates were used to investigate the effects of LYC on intracellular ROS and GSH levels. In experiment 5, a total of 84 Day-5 embryos were used in three independent replicates. To investigate the effect of LYC on ROS levels and GSH levels in PA embryos treated with the control and the optimal concentration of LYC. In experiment 6, a total of 89 Day-5 embryos were used in three independent replicates. To evaluate mitochondrial function, MMP and mitochondrial activity analyses were performed. In experiment 7, a total of 54 Day-7 blastocysts were used in three independent replicates to investigate the effect of LYC on total cell number in Day-7 blastocysts. In experiment 8, a total of 244 Day-5 PA embryos from three independent replicates were used. We measured the mRNA levels of antioxidant enzyme- and apoptosis-related genes in Day-5 PA embryos.

### 2.12. Statistical Analysis

Oocyte maturation rate = (Oocytes exhibiting first polar body/Total oocytes cultured for IVM) × 100%.

Cleavage rate = (Number of cleaved cells at 48 h/Number of parthenogenetically activated oocytes) × 100%.

Blastocyst rate = (Number of blastocysts/Number of cleaved embryos) × 100%.

Fluorescence intensity was quantified from all images using ImageJ software (version 1.54). Briefly, raw images were imported, and fluorescence intensity was measured within identical regions of interest (ROIs) using the built-in measurement tools in batch mode. Using a uniform threshold, background interference was minimized.

One-way ANOVA was used for comparisons among multiple groups. Unpaired t-tests were used for two-group comparisons. GraphPad Prism 9.0 was employed for data analysis and visualization. All experiments were independently repeated at least three times, with results expressed as mean ± SD. The following criteria were used to define statistical significance: differences were considered statistically significant at *p* < 0.05 (*) and highly significant at *p* < 0.01 (**), with *p* > 0.05 regarded as not significant.

## 3. Results

### 3.1. Effects of Different Concentrations of LYC on the In Vitro Maturation of Sheep Oocytes

**Figure 1:**
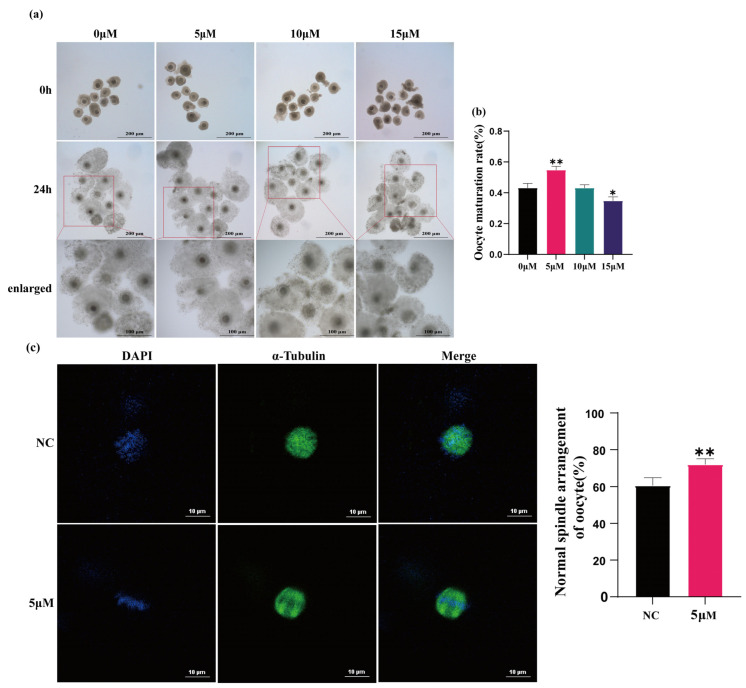
Effects of different concentrations of LYC on the in vitro maturation of sheep oocytes. (**a**) Representative images showing the growth and expansion of cumulus-oocyte complexes (COCs) under different LYC concentrations. The 5 μM LYC group exhibited the most uniform COC expansion. (**b**) Mature rate of oocytes treated with different LYC concentrations. Total number of oocytes evaluated: control, 102; 5 μM, 100; 10 μM, 98; 15 μM, 99. (**c**) Representative immunofluorescence images of spindle morphology in oocytes treated with different concentrations of LYC were determined by biomicroscopy, and the proportion of normal spindle assembly in each group was determined. Images of abnormal spindle assembly are shown for the control group, with the proportion being 37.01 ± 0.069%, whereas the 5 μM LYC group displayed images of normal spindle assembly, with the proportion being 73.23 ± 0.026%. * *p* < 0.05, ** *p* < 0.01. Results are expressed as the mean ± standard deviation; R = 3.

To determine the optimal concentration of LYC for IVM of sheep oocytes, various concentrations (0, 5, 10, and 15 μM) were evaluated. COCs’ expansion was assessed microscopically, and differences among groups were compared. The 5 μM group exhibited significantly enhanced expansion (*p* < 0.01; Figure 1a). The oocyte maturation rates in the 0, 5, 10, and 15 μM groups were 43.14 ± 2.94%, 54.67 ± 2.51%, 43.10 ± 2.10%, and 35.37 ± 2.57%, respectively. The 5 μM LYC group showed a significantly higher maturation rate than the control (*p* < 0.01; Figure 1b). We also examined spindle morphology in MII oocytes. The proportion of oocytes with normally assembled spindles was significantly higher in the 5 μM LYC group than in the control group (*p* < 0.01; Figure 1c). These findings indicate that LYC enhances IVM of sheep oocytes.

### 3.2. Effects of LYC on GSH, ROS and Mitochondrial Membrane Potential in Oocytes

**Figure 2:**
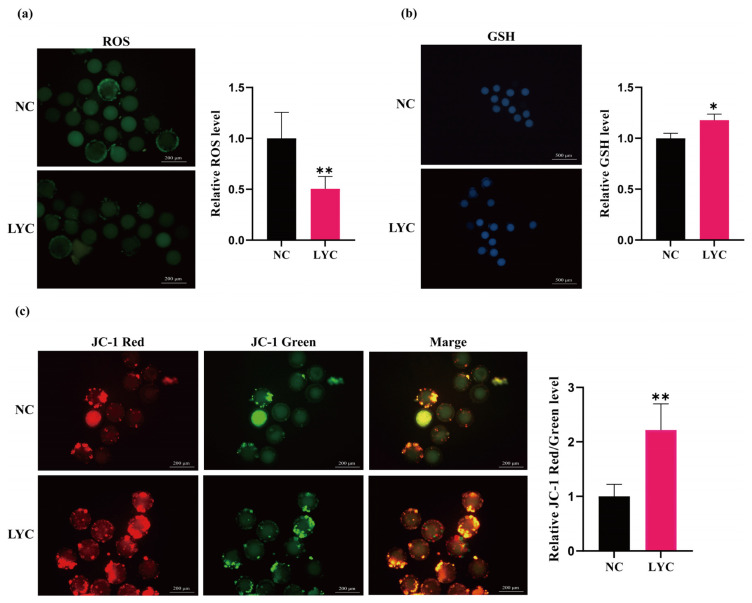
Effect of LYC on GSH, ROS and mitochondrial membrane potential in oocytes. (**a**) Intracellular ROS levels were detected using DCFH-DA staining. Treatment with 5 μM LYC significantly reduced ROS levels compared to the control group. (**b**) Intracellular GSH levels were detected using monochlorobimane staining. The 5 μM LYC group showed a marked increase in GSH levels. (**c**) MMP was assessed using JC-1 staining, expressed as the ratio of red (aggregates) to green (monomer) fluorescence. The 5 μM LYC group exhibited a significantly higher red/green ratio, indicating improved MMP. * *p* < 0.05, ** *p* < 0.01. R = 3.

To evaluate the antioxidant effect of LYC on sheep oocytes during IVM, we assessed the levels of GSH, ROS, and MMP in mature oocytes from the control and LYC-treated (5 μM LYC) groups. The addition of 5 μM LYC to the IVM culture system significantly decreased the intracellular ROS level (Figure 2a) and increased the GSH level (Figure 2b) compared with the control. Furthermore, LYC treatment markedly enhanced the mitochondrial membrane potential (Figure 2c), indicating reduced mitochondrial oxidative stress and improved mitochondrial function.

### 3.3. Effects of Different Concentrations of LYC on Early Embryonic Development

**Figure 3:**
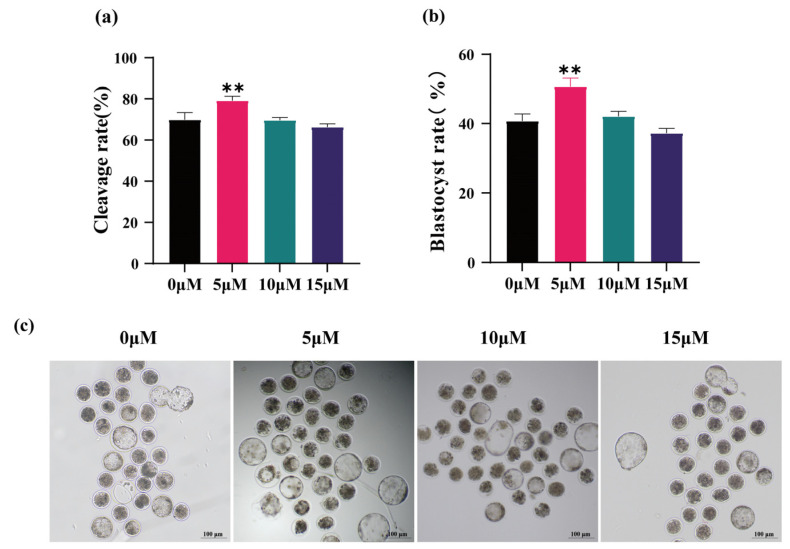
Effects of different concentrations of LYC on early embryo development in sheep (**a**) The cleavage rate for each LYC treatment group was determined after 48 h of culture. (**b**) The blastocyst rate of sheep oocytes in each LYC treatment group was counted on the 7th day. (**c**) Representative images of blastocysts under different concentrations of LYC. The total number of blastocysts evaluated for each concentration was: control, 126; 5 μM, 173; 10 μM, 164; 15 μM, 125. ** *p* < 0.01. Results are expressed as the mean ± standard deviation; R = 3.

To investigate the effects of LYC on embryo development, early embryos were cultured in vitro with varying concentrations of LYC (0, 5, 10, and 15 μM), and their cleavage and blastocyst rates were assessed. The cleavage rate for the 5 μM LYC group was significantly higher than that in the other groups (*p* < 0.01; Figure 3a). The blastocyst rate on day 7 in the 0, 5, 10, 15 μM groups was 40.73 ± 2.06%, 50.62 ± 2.49%, 42.09 ± 1.48%, and 37.27 ± 1.31%, respectively. Notably, the blastocyst rate in the 5 μM group was significantly higher than that in all other groups (*p* < 0.01; Figure 3b,c).

### 3.4. Effects of LYC on Oxidative Stress

**Figure 4:**
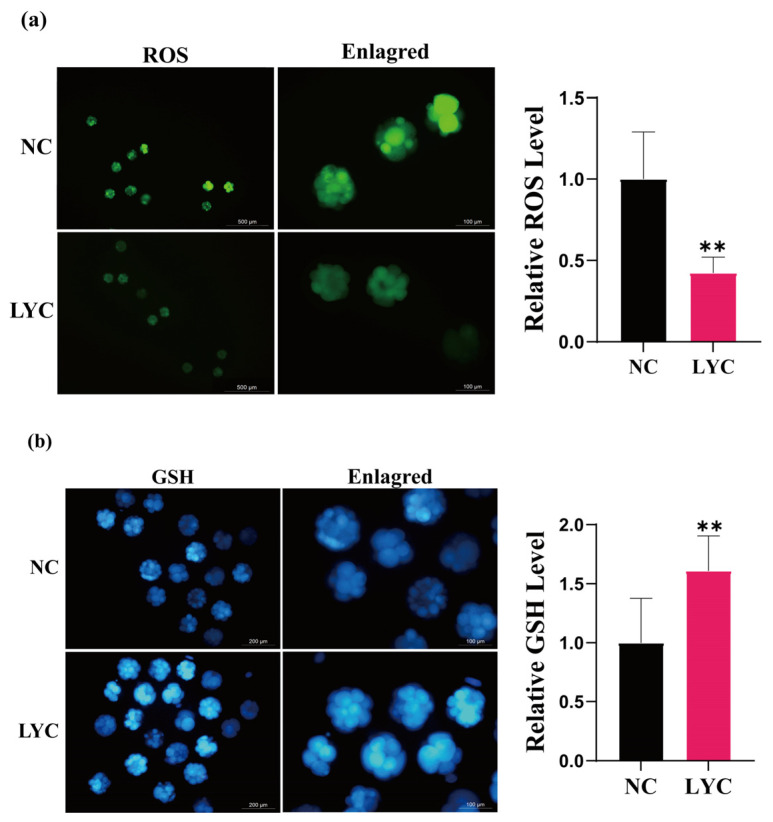
Effects of LYC on ROS and GSH levels in early ovine embryos. (**a**) Representative images and relative fluorescence intensity of ROS levels in the control and 5 μM LYC treatment groups. (**b**) Representative images and relative fluorescence intensity of GSH levels in the control and 5 μM LYC treatment groups. ** *p* < 0.01. R = 3.

The antioxidant effect of LYC on eight-cell embryos was assessed by measuring ROS and GSH levels. Compared with the control group, supplementation with 5 μM LYC during IVC significantly reduced ROS levels (*p* < 0.01; Figure 4a) and increased GSH levels (*p* < 0.01; Figure 4b) in eight-cell embryos.

### 3.5. Effect of LYC on Mitochondrial Function

**Figure 5:**
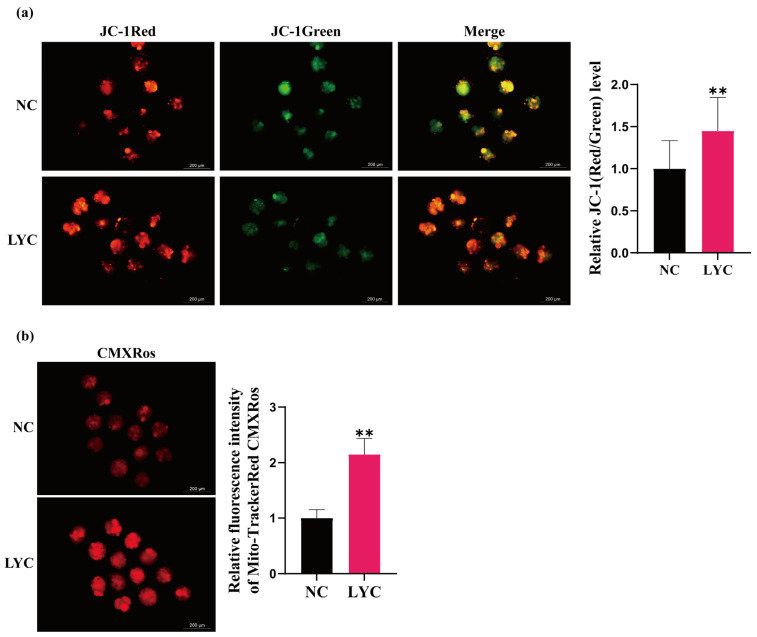
Effects of LYC on MMP and Mitochondrial Activity in Early Ovine Embryos. (**a**) Representative JC-1 staining images and the red/green fluorescence ratio, comparing MMP in control versus 5 μM LYC-treated early embryos. (**b**) Mitochondrial activity assessed by MitoTracker Red CMXRos staining in control and 5 μM LYC-treated embryos. ** *p* < 0.01. R = 3.

Mitochondrial function was assessed by measuring mitochondrial activity and the MMP in early embryos. The MMP was quantified as the ratio of red to green fluorescence intensity. Compared with the control group, early embryos treated with 5 μM LYC exhibited significantly higher MMP level (*p* < 0.01; Figure 5a) and mitochondrial activity (*p* < 0.01; Figure 5b).

### 3.6. Effects of LYC on Blastocyst Quality

**Figure 6:**
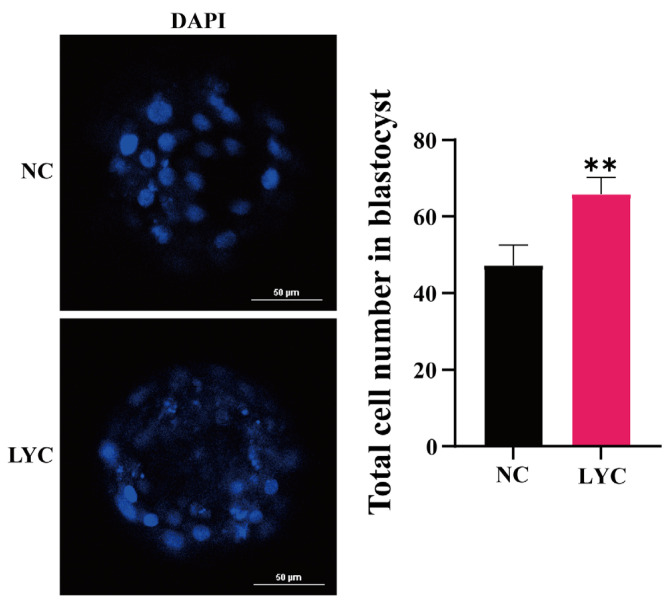
Effect of LYC on the TCN of blastocysts. Representative images of DAPI-stained blastocysts from the control and 5 μM LYC supplementation groups, along with the corresponding TCN for each group. ** *p* < 0.01. R = 3.

To investigate the effect of LYC on blastocyst quality, the total cell number of blastocysts was analyzed in the control and 5 μM LYC groups. The results demonstrated that the total cell number was significantly higher in the 5 μM LYC group compared to the control group (*p* < 0.01; Figure 6).

### 3.7. Impact of LYC on Antioxidant- and Apoptosis-Related Genes in Early Embryos

**Figure 7:**
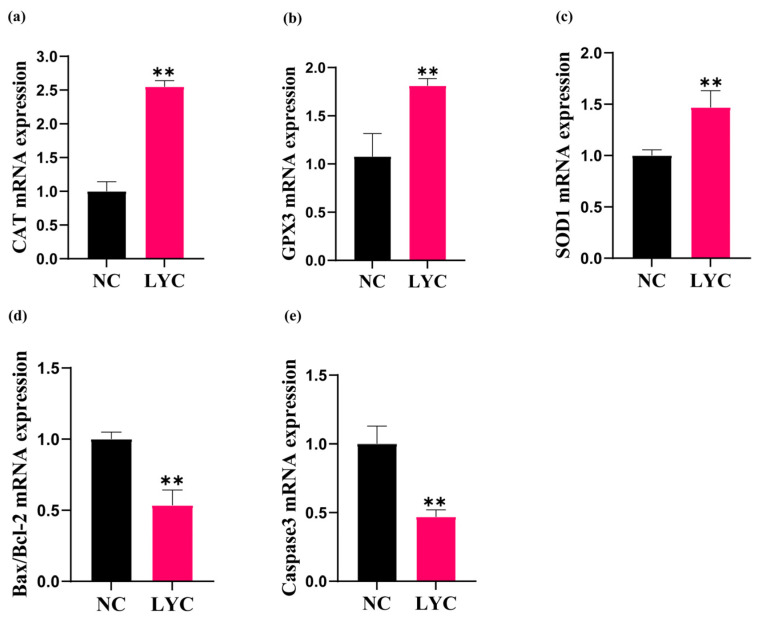
Effects of LYC on antioxidant-related genes and apoptosis-related genes in eight-cell embryos. Relative mRNA expression levels of (**a**) CAT, (**b**) GPX3, (**c**) SOD1, (**d**) the *Bax*/*Bcl-2* ratio and (**e**) *Caspase-3* were measured. ** *p* < 0.01. R = 3.

To evaluate the effects of LYC on the Expression of antioxidant and anti-apoptotic genes during in vitro development of early sheep embryos. The expression levels of the antioxidant genes *CAT*, *SOD1*, and *GPX3* were significantly higher in the 5 μM LYC group than in the control group (*p* < 0.01; Figure 7a–c). Furthermore, the *Bax*/*Bcl-2* mRNA ratio and *Caspase-3* expression levels were significantly lower in the 5 μM LYC group (*p* < 0.01; Figure 7d,e).

## 4. Discussion

The research focus of IVP lies in optimizing the in vitro culture environment for oocytes and early embryos. Although the production of high-quality embryos is essential for enhancing reproductive efficiency in sheep, the in vitro environment exposes oocytes and early embryos to elevated levels of ROS, thereby disrupting their redox homeostasis. To further investigate the effects of LYC on the developmental competence of oocytes and embryos, several key parameters were evaluated. The results showed that supplementation with 5 μM LYC during IVM positively affected the developmental potential of ovine oocytes and early embryos.

LYC was first isolated from tomatoes and is also found in various other fruits and vegetables, including watermelon and grapes [22]. LYC exhibits antioxidant activity in vivo and in vitro, which has led to its widespread use in dietary supplements, cosmetics, and pharmaceuticals [23]. LYC enhances PINK1/Parkin-mediated mitophagy, thereby helping to maintain mitochondrial homeostasis [24]. Furthermore, studies have demonstrated that LYC scavenges excess ROS during IVM and IVC, thereby preventing OS [18]. In this study, 5 μM LYC was identified as the most effective concentration among those tested for promoting granulosa cell proliferation. CCs are intimately associated with the oocyte, providing the nutrients and energy required for oocyte maturation and subsequent early embryonic development.

Compared with the other treatment groups, supplementation with 5 μM LYC significantly enhanced the maturation rate of ovine oocytes and the subsequent blastocyst rate. Furthermore, supplementation with 5 μM LYC during IVC markedly reduced intracellular ROS levels while increasing GSH content. During in vitro embryo production, the excessive accumulation of intracellular ROS can cause irreversible damage to oocytes and embryos [25]. GSH plays a key role in reducing intracellular ROS levels in mature oocytes and early embryos [26]. The balance between intracellular ROS and GSH levels is a critical determinant of the developmental competence of mature oocytes and early embryos [27]. In addition, GSH is essential for the progression of mature oocytes and early embryos to the blastocyst stage [28]. This study demonstrated that supplementation with 5 μM LYC significantly increased GSH levels during the development of mature oocytes and early embryos, a finding consistent with previous reports.

The mitochondrial cristae house the protein complexes of the oxidative phosphorylation mechanism, and ROS are generated as a byproduct of this process [29]. Studies have shown that mitochondrial dynamics play a critical role in energy production, regulating the changing energy demands of oocytes and early embryos and thereby influencing their developmental quality [30]. During in vitro production of mature oocytes and embryos, excessive ROS accumulation induces enhanced mitophagy and mitochondrial dysfunction [31]. Zhang et al. [32] found that the addition of rutin during oocyte development enhanced the developmental potential of sheep GV-stage oocytes by reducing mitophagy. Studies have shown that MitoQ enhances the developmental competence of porcine oocytes and embryos by reducing oxidative stress and improving mitochondrial function [33]. Wang et al. [34] found that LYC treatment activated Nrf2 nuclear translocation and the downstream antioxidant signaling pathways of HO-1 and NQO1; this consequently alleviated mitochondrial damage. In this study, LYC inhibited intracellular ROS generation, increased the mitochondrial membrane potential, and enhanced mitochondrial activity. These results indicate that LYC enhances the developmental competence of ovine oocytes and early embryos through the regulation of mitochondrial function.

Ji et al. [35] found that the addition of Mitoquinone mesylate during the embryonic development of Tibetan sheep suppressed H_2_O_2_-induced apoptosis by inhibiting oxidative stress. Studies have shown that the addition of LYC during early embryonic development reduces the expression of CASP3 and BAX, increases the expression of BCL2, and consequently lowers the apoptosis rate in early embryos [36]. This is critical for embryonic development, as the relevant proteases in all apoptotic pathways are regulated by the BCL2 protein family [37]. This is consistent with our findings, in which the treatment with 5 μM LYC in the experimental group demonstrated a reduction in the expression of the pro-apoptotic factor CASP3 and in the BAX/BCL2 ratio.

In this experiment, PA was employed using embryos that possessed only maternal genetic material to observe the embryonic development process and to better investigate the effects of LYC on early ovine embryonic development. IVP is a key technological approach in modern livestock production for enhancing the reproductive efficiency of superior livestock and accelerating genetic improvement. Studies have demonstrated that supplementing with LYC during early porcine embryonic development increases the inner cell mass count, reduces ROS levels, and decreases cell apoptosis, thereby enhancing the developmental potential of porcine embryos [38]. This study demonstrates that supplementing with 5 μM LYC during the IVM of ovine oocytes significantly increases the ICM cell count in blastocysts, indicating that LYC enhances the developmental competence of early embryos.

We must acknowledge that this in vitro study has certain limitations. Although our results demonstrate that supplementation with LYC during IVM improves oocyte quality and subsequent embryo development, the lack of systemic metabolic, hormonal, and immune regulation in the in vitro environment means that these findings may not be directly extrapolated to in vivo conditions. Therefore, future studies are warranted to further validate the protective effects of LYC through in vivo experiments.

## 5. Conclusions

In conclusion, these results demonstrate that LYC improves mitochondrial function in oocytes and early embryos. Furthermore, LYC regulates oocyte and early embryo apoptosis and alleviates oxidative damage by increasing GSH levels and reducing ROS levels. These findings not only extend the oocyte and embryo protective effects of LYC to sheep, a ruminant species, but also provide a novel, cost-effective nutritional intervention strategy to improve the efficiency of in vitro embryo production in high-quality sheep, thereby advancing knowledge on the regulation of the maternal-to-embryonic transition in livestock reproductive biotechnology.

## Figures and Tables

**Table 1:** Primer sequences for qRT-PCR.

Genes	Primer Sequence (5′-3′)	GenBank Code	Size (bp)
*CAT*	F: CCAGCGACCAGATGAAAC R: CGGTCAAAGTGAGCCATT	XM_004016396	175
*GPX3*	F: CCATTCGGTCTGGTCATT R: CCCGTTCACATCGCCTTT	XM_015096153	156
*SOD1*	F: AGGGAGATAAAGTCGTCGTA R: ACAGAGGATTAAAGTGAGGG	NM_001145185	129
*Bax*	F: CATGGGCTGGACATTGGACT R: CCAGATGGTGAGTGAGGCAG	XM_027978594.2	157
*Bcl-2*	F: TGGCCTTCTTTGAGTTCGGA R: CGGTTCAGGTACTCGGTCAT	XM_027960877.2	106
*Caspase3*	F: TCAGGGAAACCTTCACGAGC R: ATCGACGGGTCCATTGGTTC	XM_060406953.1	185
*β-Actin*	F: AGATTATCGCTCCTCCCG R: CTCATCATACTCCTGCTTGCT	XM_004013078.5	110

## Data Availability

The original contributions presented in this study are included in the article. Further inquiries can be directed to the corresponding author.

## Abbreviations

The following abbreviations are used in this manuscript:

LYC	Lycopene
ROS	Reactive Oxygen Species
GSH	Glutathione
MMP	Mitochondrial Membrane Potential
FSH	Follicle Stimulating Hormone
LH	Luteinizing Hormone
OS	Oxidative Stress
